# Response shift, recall bias and their effect on measuring change in health-related quality of life amongst older hospital patients

**DOI:** 10.1186/1477-7525-8-65

**Published:** 2010-07-10

**Authors:** Steven McPhail, Terry Haines

**Affiliations:** 1 Centre for Functioning, Disability and Health Research, Queensland Health, Buranda Plaza, Corner of Ipswich Road and Cornwall Street, Brisbane, Australia; 2 School of Public Health and Institute of Health and Biomedical Innovation, Queensland University of Technology, Kelvin Grove, Australia; 3 The University of Queensland, School of Health and Rehabilitation Sciences, St Lucia, Australia; 4 Southern Health, Allied Health Clinical Research Unit, Kingston Centre, Cnr Warrigal and Kingston Roads, Cheltenham, Australia; 5 Monash University, Physiotherapy Department, School of Primary Health Care, Monash University Peninsular Campus, Victoria, Australia

## Abstract

**Background:**

Assessments of change in subjective patient reported outcomes such as health-related quality of life (HRQoL) are a key component of many clinical and research evaluations. However, conventional longitudinal evaluation of change may not agree with patient perceived change if patients' understanding of the subjective construct under evaluation changes over time (response shift) or if patients' have inaccurate recollection (recall bias). This study examined whether older adults' perception of change is in agreement with conventional longitudinal evaluation of change in their HRQoL over the duration of their hospital stay. It also investigated this level of agreement after adjusting patient perceived change for recall bias that patients may have experienced.

**Methods:**

A prospective longitudinal cohort design nested within a larger randomised controlled trial was implemented. 103 hospitalised older adults participated in this investigation at a tertiary hospital facility. The EQ-5D utility and Visual Analogue Scale (VAS) scores were used to evaluate HRQoL. Participants completed EQ-5D reports as soon as they were medically stable (within three days of admission) then again immediately prior to discharge. Three methods of change score calculation were used (conventional change, patient perceived change and patient perceived change adjusted for recall bias). Agreement was primarily investigated using intraclass correlation coefficients (ICC) and limits of agreement.

**Results:**

Overall 101 (98%) participants completed both admission and discharge assessments. The mean (SD) age was 73.3 (11.2). The median (IQR) length of stay was 38 (20-60) days. For agreement between conventional longitudinal change and patient perceived change: ICCs were 0.34 and 0.40 for EQ-5D utility and VAS respectively. For agreement between conventional longitudinal change and patient perceived change adjusted for recall bias: ICCs were 0.98 and 0.90 respectively. Discrepancy between conventional longitudinal change and patient perceived change was considered clinically meaningful for 84 (83.2%) of participants, after adjusting for recall bias this reduced to 8 (7.9%).

**Conclusions:**

Agreement between conventional change and patient perceived change was not strong. A large proportion of this disagreement could be attributed to recall bias. To overcome the invalidating effect of response shift (on conventional change) and recall bias (on patient perceived change) a method of adjusting patient perceived change for recall bias has been described.

## Background

Measurement of change in patient outcomes is important when evaluating the effect of health interventions or disease processes on an individual or group[1]. Objective tests of patient body, structure or function can be simple (e.g. blood pressure) or complex (e.g. positron emission tomography). These are widely used, and can provide insights essential for ongoing patient management. However, not all health constructs of importance can be measured using objective measures such as these[2]. Constructs such as pain, fatigue, depression and ultimately health-related quality of life can be just as important (if not more so) for evaluating treatment effect in some conditions. However, these constructs generally need to be measured using more subjective approaches[2,3]. Increasingly, funding decisions in health care are being based upon whether particular health programs or diseases impact upon subjectively measured outcomes such as these[2-4].

A conventional approach to evaluation of change in self-reported outcomes involves completion of a standardised measurement instrument at a certain time point (e.g. pre-treatment) and then again at a later time point (e.g. post treatment)[3,5]. Conventional measurement of change in the self-reported outcome involves subtracting the pre-treatment from the post-treatment assessment. While this approach seems logical, a momentous confounding factor may exist. If through any number of mechanisms and internal processes, a patients' understanding or perception of the construct under evaluation changes over time then comparison of two longitudinal assessments may be analogous to comparison of the proverbial apple and orange. This change in perception has been given the term 'response shift'[6-9]. If response shift occurs between assessments it is entirely likely, that patients will disagree with the magnitude and possibly the direction of conventional change score calculations[6,8,9]. The scenario presented below (Scenario 1) illustrates a change in perception that a patient may have experienced when reporting their health-related quality of life using a simple zero to ten scale.

### Scenario 1 - A patient visits his doctor six months after undergoing a prostate resection

Dr: "Tell me Bill (patient), how are you? Tell me on a scale from zero to ten where zero is the worst health you can imagine and ten is the best."

Patient: "Well I'm having a lot of trouble so I would give myself five out of ten."

Dr: "Are you better or worse than how you were six months ago?"

Patient: "When I think back about how I was feeling six months ago, I would give myself a nine out of ten because I wasn't this bad."

Dr: "What did you think at the time? Can you remember what you told me six months ago?"

Patient: "I'm not sure, I remember you asking me, I think I said six out of ten but I didn't know then how bad the symptoms could get."

Dr: "Let me check your file. Here, when I asked you six months ago you actually gave yourself a three out of ten. I made notes here about your pain, your difficulty urinating, and how anxious this was making you feel."

Patient: "Oh, yes, now I remember. I had forgotten about how anxious I was before, but the other symptoms are worse now. So, doctor, are you saying I'm better now than I was back then?"

Dr: "You tell me?"

Conflicting change scores may be calculated from this patients' report. Conventional change score calculation would compare the patients' current report (5/10) to their previous report (3/10) and infer an improvement of two points. Philosophically, if we use this calculation as the measure of change, we imply that the most appropriate perspective from which to rate a health state is the perspective held at the time of the assessment. However, this does not consider how an individual's perception of the construct under evaluation, in this case health-related quality of life, might have changed between measurement points (i.e. response shift). It is also possible to calculate patient perceived change by comparison of the current report (5/10) with their current perception of how they would rate how they were feeling previously (9/10) and infers a reduction of four points. If we use this calculation as the measure of change, we imply that the most appropriate perspective from which to rate a health state is the perspective held at one point in time. An advantage of adopting this view is that changing standards of self-assessment over time are eliminated from the calculation of change. Retrospective reporting of a construct such as this from the patient's current perspective has been termed a 'then test.'[10,11] Then tests are the most commonly reported method of assessing patient perceived change in self-reported outcomes such as health-related quality of life and fatigue to indicate whether response shift has occurred[10-12].

While the 'then test' is useful in revealing the patient's current perception of change and is amenable to use in clinical assessments, it is potentially confounded by recall bias[12]. A patient may not be able to accurately recall their health in relation to the evaluation process at a previous assessment and may remember rating their health as being better or worse than they previously did. Again consider Scenario 1. The patient recalled previously rating their health-related quality of life as 6/10, despite actually rating it as 3/10 at the initial assessment as the patient had forgotten how anxious they were feeling at the time. This three point difference due to imperfect recall would bias a patient's currently perceived change. Thus a third approach to calculating change would be to adjust the patient's currently perceived change for their recall bias. For our scenario in Scenario 1, the patient's recall bias was +3 and the patient perceived change was -4, resulting in a final change score of -1.

The three potential change scores are represented by the following equations:

Despite the potentially invalidating consequences of inaccurate representations of change in patient reported outcomes, there have been few empirical investigations providing evidence to inform discussion around this issue. Evidence supporting the existence of response shift amongst various individual patient groups has been reported,[6,10,13,14] although it has been concluded that recall bias may have influenced retrospective assessments of change, such as use of the then-test, to evaluate the magnitude and direction of response shift observed[12,13,15]. Along this line of investigation, a recently study reported poor agreement between conventional change and patient perceived change in health-related quality of life amongst a population of older adults[16]. This investigation highlighted the need to take recall bias into account during investigations of patients' perception of change in their health-related quality of life[16]. No investigation has been made to examine the potential impact of response shift and recall bias simultaneously. This study aims to investigate agreement and systematic differences between conventional change and patient perceived change as well as between conventional change and patient perceived change adjusted for recall bias in health-related quality of life amongst a group of older patients accessing healthcare resources.

## Methods

### Design

Prospective cohort investigation.

### Participants and setting

This investigation included a sample of 103 participants taking part in larger randomised controlled trial at a tertiary hospital in Brisbane, Australia. The larger trial investigated a multi-media patient education program aimed to prevent in-hospital falls amongst hospitalised older adults[17]. The participants in this investigation included a convenience sample of those who were considered by clinical staff to be likely to require a period of subacute in-hospital rehabilitation prior to discharge (with a length of stay greater than two weeks). Patients with moderate to severe cognitive deficits (e.g. Mini-Mental State Examination[18] < 24/30 or any patient in post-traumatic amnesia) were excluded as were participants with moderate or severe language deficits (e.g. aphasic stroke patients).

This patient group was selected for this investigation for several reasons. First, inpatient rehabilitation amongst hospitalised older adult groups is often focused on improving function to maximise health-related quality of life (rather than a curative effect). Therefore, meaningful evaluation of health-related quality of life is very important amongst this patient group. Additionally, due to the nature of inpatient, multi-faceted and multi disciplinary clinical interventions required, healthcare for this group is resource intensive further heightening the need for accurate and meaningful evaluation of effect. Lastly, due to health events, social changes, peer comparisons and the hospitalisation experience, patients in this group are likely to have experienced adaptation and changes in internal value systems which have lead to a response shift, particularly in regard to reporting their health-related quality of life at the beginning in comparison to the end of their hospitalisation experience.

### Measures

Health-related quality of life was evaluated using the EQ-5D instrument[19]. The first 5 questions from the EQ-5D investigate the domains of mobility, usual activities, personal care, pain/discomfort and anxiety/depression. For each of these questions the respondent may choose one of three statements indicating they either have no problems, some problems or extreme problems in that domain. A multi-attribute utility score (utility) where death and perfect health are represented by 0 and 1 respectively was calculated from these five questions by applying the Dolan tariff system[20]. Scores less than 0 are considered worse than death and 1 is the maximum score possible. The sixth and final question is an overall health state visual analogue scale (VAS) where worst imaginable and best imaginable health are represented by 0 and 100 respectively[19]. Both the utility and VAS scores were used in this investigation.

For the purpose of calculating conventional change in health-related quality of life over the length of admission, patients completed the EQ-5D on two occasions; after admission (baseline) and immediately prior to discharge (discharge). The difference between these two scores was considered conventional change in health-related quality of life.

For the purpose of calculating patient perceived change in health-related quality of life a 'then test' was also implemented using the EQ-5D instrument at the assessment immediately prior to discharge. This involved the patient reporting how they believe their HRQoL was at the baseline assessment using the EQ-5D instrument. At the discharge assessment after completing the standard EQ-5D, patients were asked to report (from their current perspective) how they believed their health-related quality of life was at the baseline assessment (using the EQ-5D instrument).

For the purpose of calculating patient recall bias, a recall test was also completed at the discharge assessment. When completing the recall test, the patient was asked to indicate what they believed they actually reported on the EQ-5D instrument at the baseline assessment. Patients were asked to complete the recall test after completing the standard EQ-5D and the EQ-5D then test. This was the third and final time the EQ-5D instrument was used at the discharge assessment (standard EQ-5D, EQ-5D then test and EQ-5D recall test).

### Procedure

All participants completed a baseline assessment that included the standard EQ-5D as soon as they were deemed medically stable by clinical staff and were able to provide written informed consent (within 72 hours of admission). Participants then completed the standard EQ-5D, EQ-5D then test and EQ-5D recall test at their discharge assessment immediately prior to discharge from the hospital. Length of stay in hospital and hence length of time between assessments was different for each patient. However, 'then test' and 'recall tests' were completed at the discharge assessment with the reference point always being their initial baseline assessment. Participants provided written informed consent prior to participation. Ethical approvals were granted by the Princess Alexandra Hospital Human Research Ethics Committee and The University of Queensland Medical Research Ethics Committee.

### Data Analysis

Demographic information including mean age, baseline and discharge health-related quality of life reports were tabulated (Table 1). Change scores were calculated for both EQ-5D utility and VAS. Conventional change scores were calculated by subtracting the baseline assessment from the discharge assessment. Patient perceived change scores were calculated by subtracting 'then test' scores from the baseline assessment. Patient perceived change adjusted for recall bias was calculated by first calculating the recall bias, then adjusting the patient perceived change by the recall bias amount. To calculate recall bias the baseline assessment was subtracted from the recall test score.

**Table 1:** Demographic information for participants included in analysis.

	Hospitalised older adults (n = 103)
Datasets complete and included in analysis (% of total)	101 (98.1%)
Length of stay in days - median (IQR)	38 (20-60)
Age - mean (sd)	73.3 (11.2)
Female - number (% of those patients included in analysis)	48 (47.5%)
Baseline health-related quality of life (EQ-5D utility) - mean (sd)	0.368 (0.338)
Baseline health-related quality of life (EQ-5D VAS) - mean (sd)	63.2 (17.1)
Perception of baseline at discharge (EQ-5D utility then test) - mean (sd)*	0.215 (0.406)
Perception of baseline at discharge (EQ-5D VAS then test) - mean (sd)*	45.7 (21.0)
Recall of baseline response (EQ-5D utility recall test) - mean (sd)*	0.231 (0.405)
Recall of baseline response (EQ-5D VAS recall test) - mean (sd)*	47.5 (20.3)
Discharge health-related quality of life (EQ-5D utility) - mean (sd)*	0.656 (0.240)
Discharge health-related quality of life (EQ-5D VAS) - mean (sd)*	72.5 (16.7)

* Collected at the discharge assessment

**Figure 1:**
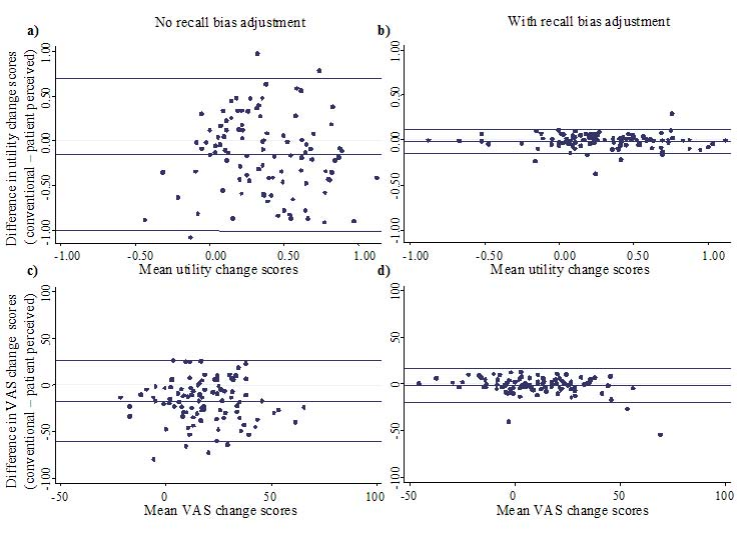
**Bland-Altman plots (with limits of agreement) for change calculated from conventional longitudinal assessments and patient perceived change in utility without (1a) and with (1b) adjustment for recall bias as well as for change in EQ-5D VAS without (1c) and with (1d) adjustment for recall bias**.

Agreement between conventional change and patient perceived change as well between conventional change and patient perceived change adjusted for recall bias were calculated using intraclass-correlation coefficients and limits of agreement (separately for utility and VAS). To evaluate whether any systematic difference existed (i.e. whether conventional change was consistently higher or lower than patient perceived change or patient perceived change adjusted for recall bias), paired t-tests were employed (Table 2). Bland-Altman plots with limits of agreement [21] were also prepared (Figure 1) to visually represent agreement levels between conventional change and patient perceived change as well as for conventional change and patient perceived change adjusted for recall bias (for EQ-5D utility and VAS).

**Table 2:** Mean change, intraclass correlation coefficient (ICC), and limits of agreement (LOA) between change scores calculated from conventional longitudinal assessments and the patients' perspective (with and without adjustment for recall bias).

Measure	Patient perspective adjusted for recall bias	Conventional change mean (95% CI)	Patient perspective change mean (95%CI)	ICC (95% CI)	Limits of agreement			p-value*
						
					Lower LOA (95% CI)	Mean difference (95% CI)	Upper LOA (95% CI)	
EQ-5D Utility	No	0.287 (0.216,0.359)	0.441 (0.367,0.518)	0.34 (0.16,0.50)	-1.007 (-1.092,-0.922)	-0.150 (-0.239,-0.069)	0.700 (0.616,0.785)	< 0.001*
EQ-5D Utility	Yes	0.287 (0.216,0.359)	0.303 (0.232,0.375)	0.98 (0.97,0.99)	-0.150 (-0.163,-0.136)	-0.016 (-0.116,-0.084)	0.118 (0.105,0.131)	0.019*
EQ-5D VAS	No	9.3 (5.4,13.2)	26.7 (22.8,30.7)	0.40 (0.22,0.55)	-60.7 (-65.0,-56.4)	-17.4 (-21.7,-13.1)	25.8 (21.5,30.1)	< 0.001*
EQ-5D VAS	Yes	9.3 (5.4,13.2)	11.0 (6.7,15.3)	0.90 (0.86,0.93)	-19.9 (-21.8,-18.1)	-1.7 (-3.5,0.1)	16.5 (14.7,18.3)	0.060

* A p-value < 0.05 indicates that a systematic difference exists (i.e. change from patient perspective was either consistently higher or consistently lower than conventional change scores.

To examine the magnitude of discrepancy between change scores within individuals, the absolute difference between conventional change and patient perceived change was calculated for each participant (regardless of direction). To assist interpretation of whether the magnitude of difference between these change scores within individuals was clinically meaningful, the number of participants with a discrepancy between utility change scores greater than a minimal clinically important difference of 0.081 was calculated (Table 3). This value (0.081) was previously reported as the median value for EQ-5D utility minimal clinically important difference from a review of 8 investigations incorporating 11 populations[22]. In the same way the absolute difference between conventional change and patient perceived change adjusted for recall bias was also calculated for each participant, and the number with a discrepancy in utility greater than 0.081 was also calculated (Table 3).

**Table 3:** Absolute differences between conventional and patient perceived change (with and without adjustment for recall bias) and the number of patients with this difference greater than a minimal clinically important difference (MCID) in utility of 0.081.

	Utility mean (sd)	VAS mean (sd)	> MCID number (%)
No adjustment for recall bias	0.363 (0.273)	22.3 (16.6)	84 (83.2%)
With adjustment for recall bias	0.021 (0.066)	3.5 (8.6)	8 (7.9%)

To specifically investigate the effect of recall bias on patient perceived change, agreement between patient perceived change with and without adjustment for recall bias was also calculated using intraclass-correlation coefficients and limits of agreement (Table 4). To evaluate whether any systematic difference existed (i.e. whether adjusting for recall bias resulted in consistently higher or lower patient perceived change scores), paired t-tests were employed (Table 4).

**Table 4:** Mean change, intraclass correlation coefficient (ICC), and limits of agreement (LOA) between change scores calculated from the patients' perspective with adjustment for recall bias and from the patients' perspective without adjustment for recall bias.

Measure	Patient perspective of change		ICC (95% CI)	Limits of agreement			p-value*
				
	With recall bias adjustment mean (95% CI)	Without recall bias adjustment mean (95% CI)		Lower LOA (95% CI)	Mean difference (95% CI)	Upper LOA (95% CI)	
EQ-5D Utility	0.303 (0.232,0.375)	0.441 (0.367,0.518)	0.36 (0.18,0.52)	-0.979 (-1.063,-0.895)	-0.138 (-0.221,-0.054)	0.704 (0.620,0.787)	0.002*
EQ-5D VAS	11.0 (6.7,15.3)	26.7 (22.8,30.7)	0.50 (0.34,0.64)	-57.1 (-61.2,-53.0)	-15.7 (-19.8,-11.6)	25.7 (21.6,29.8)	< 0.001*

*A p-value < 0.05 indicates that a systematic difference exists (i.e. patient perspective of change adjusted for recall bias was consistently higher or consistently lower than when no adjustment for recall bias was made.

## Results

Demographic and health-related quality of life reports are presented in Table 1. Two datasets were incomplete due to the unexpected discharge of two patients from hospital (without reassessment); these two datasets were excluded from all analysis. From the baseline assessment it can be seen that health-related quality of life was low amongst this elderly, hospitalised patient group[23]. The median (inter-quartile range) for length of stay was 38 (20-60) days.

Mean change scores and agreement statistics between conventional change and patient perceived change as well as between conventional change and patient perceived change adjusted for recall bias are presented in Table 2. Intraclass correlation coefficient (ICC) statistics indicated that agreement between conventional change and patient perceived change was not strong (EQ-5D utility = 0.34, EQ-5D VAS = 0.40). This was consistent with the limits of agreement statistics and Bland-Altman plots (Figure 1a and 1c) which covered a large proportion of the possible change scores. After adjusting patient perceived change for recall bias, ICC statistics (EQ-5D utility = 0.98, EQ-5D VAS = 0.90), limits of agreement and Bland-Altman plots (Figure 1b and 1d) indicated that agreement with conventional change was much stronger. The mean patient perceived change was greater than mean conventional change scores for both utility and VAS. Although this mean difference was statistically significant with and without adjustment for recall bias, the magnitude of the mean difference only exceeded reported minimal values for clinically important difference when no adjustment for recall bias was made (Table 2)[22].

The absolute difference between conventional change scores and patient perceived change score (with and without adjustment for recall bias) are presented in Table 3. Within individuals, discrepancy between conventional longitudinal change and patient perceived change was considered clinically meaningful for 84 (83.2%) of participants, after adjusting for recall bias this reduced to 8 (7.9%).

Agreement between patient perceived change scores with and without adjustment for recall bias was not strong. Agreement statistics for this relationship are presented in Table 4. Intraclass correlation coefficients did not indicate strong agreement for either EQ-5D utility (ICC = 0.36) or EQ-5D VAS (ICC = 0.50). This was consistent with the limits of agreement, which covered a large proportion of possible responses (Table 4). Mean patient perceived change in EQ-5D utility and VAS was less positive after adjustment for recall bias (p = 0.002 and p < 0.001 respectively) with the size of this difference large enough to be considered clinically meaningful (Table 4)[22].

## Discussion

### Main findings

Serious undesirable consequences may result from inaccurate representation of change in self-reported patient health states. This investigation has indicated that agreement between conventional change and patient perceived change in health-related quality of life, as evaluated with the 'then test,' was not strong (agreement coefficient levels below 0.40 are considered indicative of poor agreement)[24-26]. Additionally, mean conventional change scores were significantly lower than patient perceived change scores (Table 2 and Figure 1), this difference was large enough to be considered clinically meaningful[22]. Within individuals the disagreement between conventional longitudinal change and patient perceived change was substantial with 83.2% of individuals reporting a discrepancy great enough to be considered clinically meaningful (Table 3).

After adjusting patient perceived change for recall bias the agreement with conventional change was much stronger (Table 2 and Figure 1). The mean difference also diminished to a level below that which is likely to be considered clinically meaningful difference[22]. Furthermore after adjustment for recall bias, agreement between patient perceived change and conventional change was much stronger within individuals, with only 7.9% reporting a discrepancy large enough to be considered clinically meaningful (Table 3). Adjusting patient perceived change for recall bias resulted in less positive reports of change in both EQ-5D utility and VAS (Table 4).

The pattern of main findings described above indicate that amongst this patient sample over the duration of their hospital stay, a large proportion of the disagreement between patient perceived change and conventional longitudinal change could be attributed to recall bias rather than response shift. While this was the case during the investigation at hand, the relative contribution of response shift and recall bias may vary across other patient groups and amongst this type of population in other circumstances (such as the transition from hospital to the community). Response shift has the potential to invalidate conventional change scores while recall bias has the potential to invalidate patient perceived change measured using retrospective reports, such as the then-test. This investigation has been the first to incorporate a method of adjusting patient perceived change for patient recall bias.

### Wider implications

For an individual patient, inappropriate implementation, continuation or cessation of a health intervention may occur if a decision is reached based on clinical reasoning flawed by inaccurate representations of change in a relevant self-reported outcome. Perhaps of even greater consequence, evaluation of the effectiveness of a certain health intervention during a randomised trial may be compromised if one group experiences a systematic response shift[27]. In this investigation the mean conventional change was statistically lower and than the mean patient perceived change (even after adjustment for recall bias) implying that a systematic response shift (albeit very small in this case) had occurred. If during a randomised trial, a systematic response shift of a clinically important magnitude occurred due to the nature of an intervention, inappropriate conclusions regarding effect on health-related quality of life may be drawn. Furthermore other clinically important patient reported outcomes such as pain, fatigue and anxiety, may be affected.

Consider a trial examining a certain experimental surgery designed to reduce rheumatic pain in comparison to conventional conservative management. It is possible that patients in the surgery group may experience a very painful and prolonged post-operative recovery period, which could result in a response shift in relation to their pain rating. If this were to occur, conventional post - pre evaluation of pain ratings may imply a reduction in pain despite individuals not actually feeling any less pain then they did prior to the surgery. A false positive result such as this is likely to lead to further investigations of the technique that may also report similar results and ultimately superfluous adoption of a potentially harmful intervention[27]. Economic evaluation of health interventions may also be invalidated if a similar effect resulted in an inaccurate representation of change in health-related quality of life that was subsequently used in a cost-utility analysis.

The method of adjustment reported in this investigation has the potential to highlight invalidating effects of response shift and recall bias as well as offering an alternate method for change score calculation. A response shift and recall bias sensitivity analysis could be conducted to examine whether the different methods of change score calculation affects conclusions drawn in clinical trials. If the same conclusions would be drawn regardless of whether conventional or adjusted change scores were used, this may indicate that results were robust against response shift and recall bias. However, further investigation and discussion regarding this proposed adjustment technique is warranted before adoption into wider use.

### Comparison to previous research

This investigation has employed a novel approach allowing for adjustment to self-reported outcomes to be made using a retrospective report (then test) adjusted for recall bias which may be replicated in both clinical and research settings in an effort to reduce the invalidating effects of response shift and recall bias. Comparison to prior research is difficult as this is the first investigation to employ an adjustment for patient recall bias when utilising a then-test approach. However, empirical evidence from this investigation is in line with conceptual models surrounding the response shift phenomenon[7,8]. Results from the then test without adjustment for recall bias reported in this investigation are also congruent with previous investigations of response shift that have not adjusted for recall bias[10,11,13,17]. The results from this investigation suggest that recall bias is likely to affect retrospective reports amongst patient groups similar to those in this sample and this should be taken into account in future investigations utilising retrospective reporting techniques such as the then test approach.

### Limitations and future directions

Direct extrapolation of these results may be limited to patient populations similar to those included in this study. Other patient groups and older adults in differing circumstances may not have responded in the same way as participants in this investigation. Furthermore, health-related quality of life was the only construct under investigation in this study and only one generic instrument (EQ-5D) was used to evaluate this construct. However, a method of adjusting patient perceived change for recall bias has been described in this investigation that may be applied amongst other population groups and clinical settings. Further empirical research along this line of investigation is warranted, as is further discussion regarding the best way for clinicians and researchers alike to discern 'real change' amongst patient reported outcomes of a subjective nature. Particularly amongst patient groups where improvement in these subjective constructs is often the ultimate aim of health interventions rather than a straightforward curative effect on a known pathology.

Another important area for future investigation and discussion is in regard to which perspective of change is the most important to various stakeholders (meaningful change as perceived by patients, their family/carers, health experts or organisations, other members of society who fund health interventions through taxes and insurance premiums etc.). Future investigation and discussion of these issues are required to maximise health outcomes for all members of society.

## Conclusions

Agreement between conventional change and patient perceived change was not strong. A large proportion of this disagreement may be attributable to recall bias. To overcome the invalidating effect of response shift (on conventional change) and recall bias (on patient perceived change) a method of adjusting patient perceived change for recall bias has been described.

## Competing interests

The authors declare that they have no competing interests.

## Authors' contributions

SM contributed to research idea conception, planning of research processes, data analysis and manuscript preparation, as well as manuscript review, appraisal and editing. TH contributed to research idea conception, planning of research processes as well as manuscript review, appraisal and editing. Both authors read and approved the final manuscript.

## Acknowledgements

Terry Haines is supported by a National Health and Medical Research Council (Australia) Career Development Award (606732).

This project was supported by a National Health and Medical Research Council (Australia) Project Grant (456097).
